# A modified quadriceps snip technique in primary total knee arthroplasty for hemophilic stiff knees: a retrospective case series

**DOI:** 10.3389/fmed.2026.1783017

**Published:** 2026-05-21

**Authors:** Kai Wang, Yuxi Huang, Qinghua Zhao, Rui Feng, Zewei Zheng, Mincong He, Qiushi Wei, Wei He, Qunqun Chen

**Affiliations:** 1 The Third Clinical Medical College, Guangzhou University of Chinese Medicine, Guangzhou, Chnia; 2 The Third Affiliated Hospital, Guangzhou University of Chinese Medicine, Guangzhou, China; 3 Guangdong Research Institute for Orthopedics and Traumatology of Chinese Medicine, Guangzhou, China

**Keywords:** case series, hemophilic, modified quadriceps snip technique, stiff knee, total knee arthroplasty

## Abstract

**Introduction:**

This retrospective case series was conducted to evaluate the effects of the modified quadriceps snip technique applied during primary total knee arthroplasty (TKA) in patients with end-stage hemophilic arthritis.

**Methods:**

A total of 9 patients (10 knees) with hemophilic stiff knees who underwent primary TKA using the modified quadriceps snip technique between January 2022 and June 2023 were retrospectively reviewed. The primary outcome was defined as the improvement in knee range of motion (ROM) from baseline to the final follow-up. Secondary outcomes included the Knee Society Score (KSS) clinical and functional scores and Hemophilia Joint Health Score version 2.1 (HJHS 2.1).

**Results:**

ROM improved significantly from 19.5 ± 11.17° preoperatively to 80 ± 11.54° postoperatively (60.50°, 95% CI: 48.29°–72.71°, p < 0.01). KSS clinical scores increased from 28.4 ± 20.46 to 70 ± 21.21 (41.60, 95% CI: 25.67–57.53, *p* < 0.01), and KSS functional scores improved from 42.25 ± 20.85 to 78 ± 25.85 (33.50, 95% CI: 18.60–48.40, *p* < 0.01). HJHS 2.1 scores decreased from 32 ± 8.65 to 18.1 ± 9.01 (13.90, 95% CI: -17.72 to -10.08, *p* < 0.01). No cases of infection, implant loosening, or revision surgery were recorded at the final follow-up, with a mean follow-up of 21 months (range: 16–34 months).

**Conclusion:**

In this case series, the modified quadriceps snip technique was associated with improvements in knee range of motion and clinical scores. These preliminary findings suggest that prospective controlled studies are still warranted to validate its clinical value.

## Introduction

Hemophilia is a rare inherited bleeding disorder caused by mutations in coagulation factor genes (1, 2). Hemophilia A is characterized by a deficiency of factor VIII, whereas hemophilia B results from a deficiency of factor IX (3). The disease primarily affects males and is clinically manifested by recurrent and persistent bleeding into joints, muscles, and soft tissues (4, 5). Some patients may also experience visceral or mucosal hemorrhage (6). Repeated hemarthroses lead to progressive cartilage destruction and ultimately hemophilic arthritis, most commonly involving the knees, ankles, and elbows, with symptoms typically worsening with age (7–9).

For end-stage hemophilic knee arthritis, total knee arthroplasty (TKA) is the mainstay of surgical treatment (10, 11). The long-term efficacy of TKA in improving knee function and relieving pain in hemophilic patients has been demonstrated in previous studies (12–14). However, important challenges remain, including inadequate surgical exposure, risk of patellar or quadriceps tendon injury, and suboptimal postoperative functional recovery (15, 16). Preliminary clinical studies have indicated that the subvastus approach with a quadriceps snip technique can partially mitigate these issues (17, 18), but postoperative knee extension lag may still occur. Knee extension lag is defined as a condition in which the active knee extension angle is smaller than the passive knee extension angle. Specifically, the knee can achieve full extension passively but cannot reach the passive extension limit through active muscle contraction. An angular difference greater than 0° is defined as knee extension lag. Clinically commonly used cutoff values are ≥ 10° or ≥ 15° of discrepancy between active and passive knee extension. Knee extension lag represents a frequent complication associated with postoperative knee range of motion following TKA. Studies have reported that the main recovery progress occurs within 6–12 months postoperatively, and often takes more than 12 months.

**FIGURE 1:**
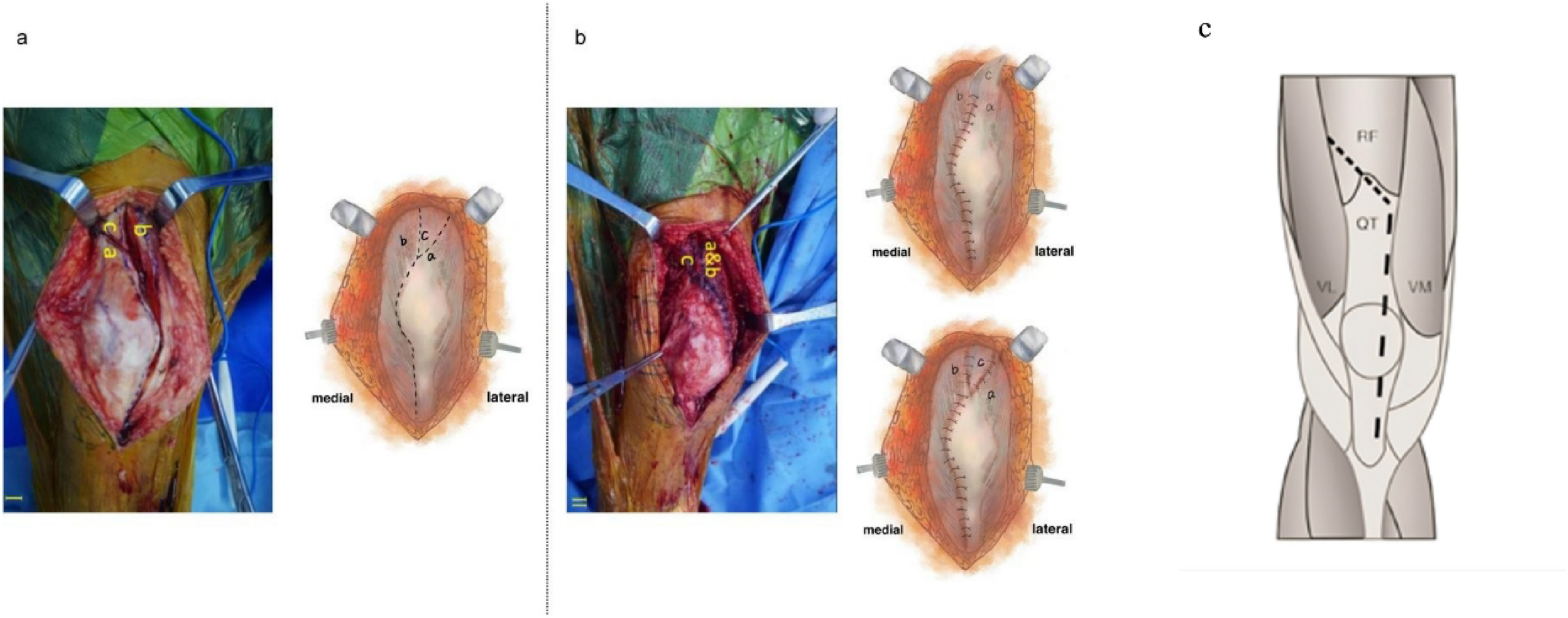
Intraoperative images. **(a)** An oblique quadriceps incision is made to expose the knee joint. Label a indicates the distal free end of the quadriceps, label b the medial aspect of the quadriceps, and label c its proximal free end. **(b)** The segment a is sutured to b; following completion of this suture, c is overlapped and sutured in place. **(c)** The quadriceps snip begins with a medial parapatellar arthrotomy and the quadriceps tendon is cut obliquely at the proximal end at a 45° angle toward the vastus lateralis. QT, quadriceps tendon; RF, rectus femoris; VL, vastus lateralis; VM, vastus medialis.

Based on the conventional quadriceps snip technique, we designed a modified technique to improve intraoperative exposure, which we refer to as the modified quadriceps snip technique. Compared with the conventional procedure, this technique involves an additional incision in the quadriceps tendon, creating a proximal free end and a distal free end. A modified quadriceps tendon suture technique, staggered suturing, was used during suturing. The medial aspect of the quadriceps tendon was first sutured to the distal free end, followed by suturing of the proximal free end. to achieve relative lengthening of the distal quadriceps tendon and avoid absolute lengthening of the tendon. The detailed procedure is illustrated in Figure 1. For comparative purposes, we also included images and descriptions of the conventional quadriceps snip technique (Figure 1c). Application of this modified technique in primary TKA for patients with end-stage hemophilic arthritis complicated by severe knee stiffness can optimize intraoperative surgical exposure and achieve a marked improvement in postoperative knee range of motion.

## Materials and methods

We retrospectively reviewed patients with hemophilic arthritis and stiff knees who underwent TKA using a modified quadriceps snip technique between January 2022 and June 2023. The inclusion criteria were as follows: (1) preoperative knee range of motion (ROM) < 50° (6); (2) all surgeries performed by a single senior surgeon; and (3) a minimum clinical follow-up of 16 months. Some studies have suggested that knee function, pain relief, and range of motion in ordinary patients reach a stable plateau approximately 12 months after TKA (19). We believe that the rehabilitation period for patients with hemophilic arthritis following TKA should be longer compared with that for ordinary patients. Therefore, we selected a follow-up duration of at least 16 months to objectively evaluate long-term outcomes and avoid bias associated with short-term follow-up. Exclusion criteria were: (1) insufficient or incomplete clinical data; and (2) irregular or loss to follow-up. We defined the endpoint of follow-up as the last available clinical and radiographic assessment for each patient. This case series was approved by the institutional ethics review board (Approval No.: PJ-XS-20240829-001).

Two independent observers assessed the preoperative and postoperative clinical status. They evaluated Knee Society Score (KSS) clinical and functional components (the clinical component includes a dedicated pain score), active ROM of the affected knee, Hemophilia Joint Health Score (HJHS) 2.1, perioperative and postoperative complications, and perioperative coagulation factor levels. Postoperative full-length lower limb radiographs were obtained to assess implant position, limb alignment, implant loosening, and laboratory examinations were combined to evaluate postoperative infection. All patients provided informed consent for participation and data use. ROM: Active range of motion was measured by two independent observers using a standard goniometer. KSS/HJHS: Assessed by two independent observers, with the mean value used for analysis.

Perioperative coagulation factor management and complication prevention are critical components of surgical care for patients with hemophilia. Preoperative evaluation included the administration of factor VIII at a standardized dose of 20 IU/kg body weight. To ensure adequate hemostatic coverage, factor VIII activity levels were systematically measured at 2 and 10 h post-administration, accompanied by routine inhibitor screening to detect potential neutralizing antibodies. Detailed perioperative coagulation factor levels and corresponding activity measurements are comprehensively presented in Table 1, providing a reference framework for surgical hemostatic management in this patient population.

**TABLE 1:** Peroperative coagulation factor levels.

Time period	Factor level (%)
Surgery day, POD 1–3	80–100
POD 4–6	40–80
POD 7–14	30–60

### Surgical technique

All surgeries were performed in a standard operation room under general anesthesia, with a pneumatic thigh tourniquet applied. Cemented knee prostheses were used in all cases. Preoperative inhibitor screening and coagulation factor testing were conducted to ensure factor activity levels > 80% on the day of surgery.

A standard midline knee skin incision was made, followed by a medial parapatellar arthrotomy and release of suprapatellar adhesions. The infrapatellar fat pad was partially excised as needed to enhance surgical exposure, while the lateral portion was preserved when possible. The modified quadriceps snip technique was then applied to improve joint exposure. The modified quadriceps snip technique was then applied to improve joint exposure (Figure 1). When necessary, a lateral retinacular release was performed to further optimize visualization. Synovectomy and osteophyte removal were undertaken, followed by standard femoral and tibial bone cuts. Particular attention was paid to avoiding injury to the collateral ligaments, common peroneal nerve, and major vascular structures. A posterior capsular release was performed to obtain adequate extension space. Patellar resurfacing was not performed in any case. Trial components were inserted to assess knee ROM and soft tissue balance, and the final cemented prosthesis was implanted once satisfactory ROM and stability were achieved.

During closure of the joint cavity, a modified quadriceps suturing technique was employed. With the knee flexed at 30° to reduce tension on and prevent injury to the patellar tendon, the proximal and distal ends of the transected quadriceps were identified and separated. Adequate dissection and release of the medial knee tendons and surrounding soft tissues were performed. Non-absorbable sutures were used to anchor the distal end of the severed quadriceps to the medial knee tendons and adjacent soft tissues (as shown in Figure 1). The proximal end of the quadriceps was then sutured to the same medial tendinous and soft tissue structures, thereby effectively lengthening the quadriceps tendon. Absorbable sutures were applied to reinforce the repair. Intraoperative testing of knee flexion and assessment of the strength of the soft tissue repair were performed thoroughly. A drainage tube was routinely placed, and the wound was closed in layers. Subcutaneous closure was performed using a cosmetic suturing technique, with additional interrupted sutures applied if required. An elastic bandage was used for compression dressing, and all patients received a patient-controlled analgesia pump postoperatively.

Continuous passive motion (CPM) exercises were initiated on the first postoperative day, with the intraoperatively achieved maximum flexion angle used as the upper limit. Considering the high bleeding risk in hemophilia patients, prolonged closed drainage was employed to reduce the formation of intra-articular hematoma and the occurrence of joint stiffness, the drainage tube was generally maintained for 7 to 10 days until drainage was minimal. Patients were encouraged to begin weight-bearing exercises on the first postoperative day and were instructed to perform ankle pump exercises and wear compression stockings to prevent venous thrombosis. None of the patients received oral anticoagulants for thrombosis prophylaxis. Antibiotics were administered for 24–72 h postoperatively to prevent infection. Postoperative analgesia consisted of intravenous tramadol or oral COX-2 inhibitors, combined with routine local ice application. The compression dressing was removed 3 days after surgery, and patients were advised to actively perform straight-leg raises and seated leg kicks to reduce the risk of postoperative knee extension lag.

### Statistical analysis

Data were analyzed using SPSS version 27.0. All continuous data followed a normal distribution (*P* > 0.05) and are presented as mean ± standard deviation. Preoperative and postoperative outcome measures were compared using paired *t*-tests, with *P* < 0.05 considered statistically significant.

## Results

Between January 2022 and June 2023, 9 patients (10 knees) with hemophilic stiff knees underwent TKA using a modified quadriceps snip (subvastus approach with a quadriceps snip) technique. All patients completed follow-up, with a mean follow-up duration of 21 months (range: 16–34 months). The preoperative clinical characteristics of the 9 patients (10 knees) are summarized in Table 2. Postoperative outcomes, including Knee Society Score (KSS) clinical and functional scores, Hemophilia Joint Health Score (HJHS) 2.1, and ROM, are presented in Table 3. All patients in this study experienced no postoperative complications, including infection, implant loosening, or the need for revision surgery.

**TABLE 2:** Demographic and preoperative clinical characteristics of patients with hemophilic stiff knees undergoing modified snip-assisted TKA.

Parameter	Value
Patients (knees)	9(10)
Gender (male/female)	9/0
Age (years)	38.9 ± 5.8
Affected side (left/right)	5/5
Hemophilia type (A/B)	8/2
BMI (kg/m^2^)	21.65 ± 4.01
Preoperative ROM (°)	19. 5 ± 11.17

**TABLE 3:** Preoperative and postoperative outcomes of hemophilic stiff knee patients undergoing modified snip-assisted TKA.

No.	KSS clinical score		KSS Functional score		HJHS 2.1 score		ROM(°)	
	Preop	Postop	Preop	Postop	Preop	Postop	Preop	Postop
1	45	71	45	80	41	16	25	80
2	35	115	60	100	38	19	35	100
3	16	31	20	90	31	18	30	80
4	56	84	50	100	20	8	20	110
5	60	71	15	70	21	11	10	40
6	8	67	65	80	23	10	30	100
7	24	68	10	35	30	16	10	80
8	6	71	60	80	45	40	5	80
9	30	69	70	70	38	22	5	60
10	4	53	50	75	33	21	25	70

KSS, Knee Society Score; ROM, Range of motion; HJHS, Hemophilia Joint Health Score.

**FIGURE 2:**
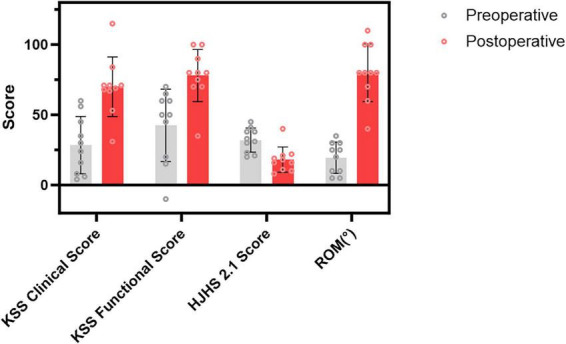
Comparison of preoperative and postoperative outcomes in hemophilic stiff knee patients undergoing modified quadriceps snip–assisted TKA.

ROM improved significantly from 19.5 ± 11.17° preoperatively to 80 ± 11.54° postoperatively (60.50°, 95% CI: 48.29°–72.71°, *p* < 0.01). KSS clinical scores increased from 28.4 ± 20.46 to 70 ± 21.21 (41.60, 95% CI: 25.67–57.53, *p* < 0.01), and KSS functional scores improved from 42.25 ± 20.85 to 78 ± 25.85 (33.50, 95% CI: 18.60–48.40, *p* < 0.01). HJHS 2.1 scores decreased from 32 ± 8.65 to 18.1 ± 9.01 (13.90, 95% CI: -17.72 to -10.08, *p* < 0.01). Follow-up results and pre- versus postoperative comparisons are summarized in Tables 3, 4 and illustrated in Figure 2.

**TABLE 4:** Comparison of preoperative and postoperative outcomes in hemophilic stiff knee patients undergoing modified snip-assisted TKA.

Outcome	Preoperative	Postoperative	*P*-value
KSS Clinical score	28.4 ± 20.46	70 ± 21.21	<0.01
KSS functional score	42.25 ± 20.85	78 ± 25.85	<0.01
HJHS 2.1 score	32 ± 8.65	18.1 ± 9.01	<0.01
ROM(°)	19.5 ± 11.17	80 ± 11.54	<0.01

### Representative case

A 32-year-old male patient diagnosed with hemophilia A and secondary hemophilic arthropathy presented with severe right knee stiffness and markedly restricted flexion, exhibiting a preoperative range of motion (ROM) of merely 20°. This profound limitation resulted from prolonged postoperative immobilization following open reduction and internal fixation for a traumatic patellar fracture. The surgical intervention involved meticulous removal of the retained internal fixation hardware, followed by implantation of a cemented Stryker total knee prosthesis system. The prosthetic configuration consisted of a size 4 femoral component, a size 3 tibial component, and an 11 mm posterior-stabilized (PS) polyethylene insert, selected to optimize joint biomechanics and functional restoration in this complex clinical scenario.

The modified quadriceps snip technique was used to obtain adequate joint exposure. Following prosthesis implantation, the quadriceps was repaired using the modified suturing technique described above. Postoperative management included compressive bandaging for 3 days, continuous passive motion (CPM) during hospitalization and for 3 months after discharge, and an active rehabilitation program emphasizing straight-leg raises and seated leg kicks.

**FIGURE 3:**
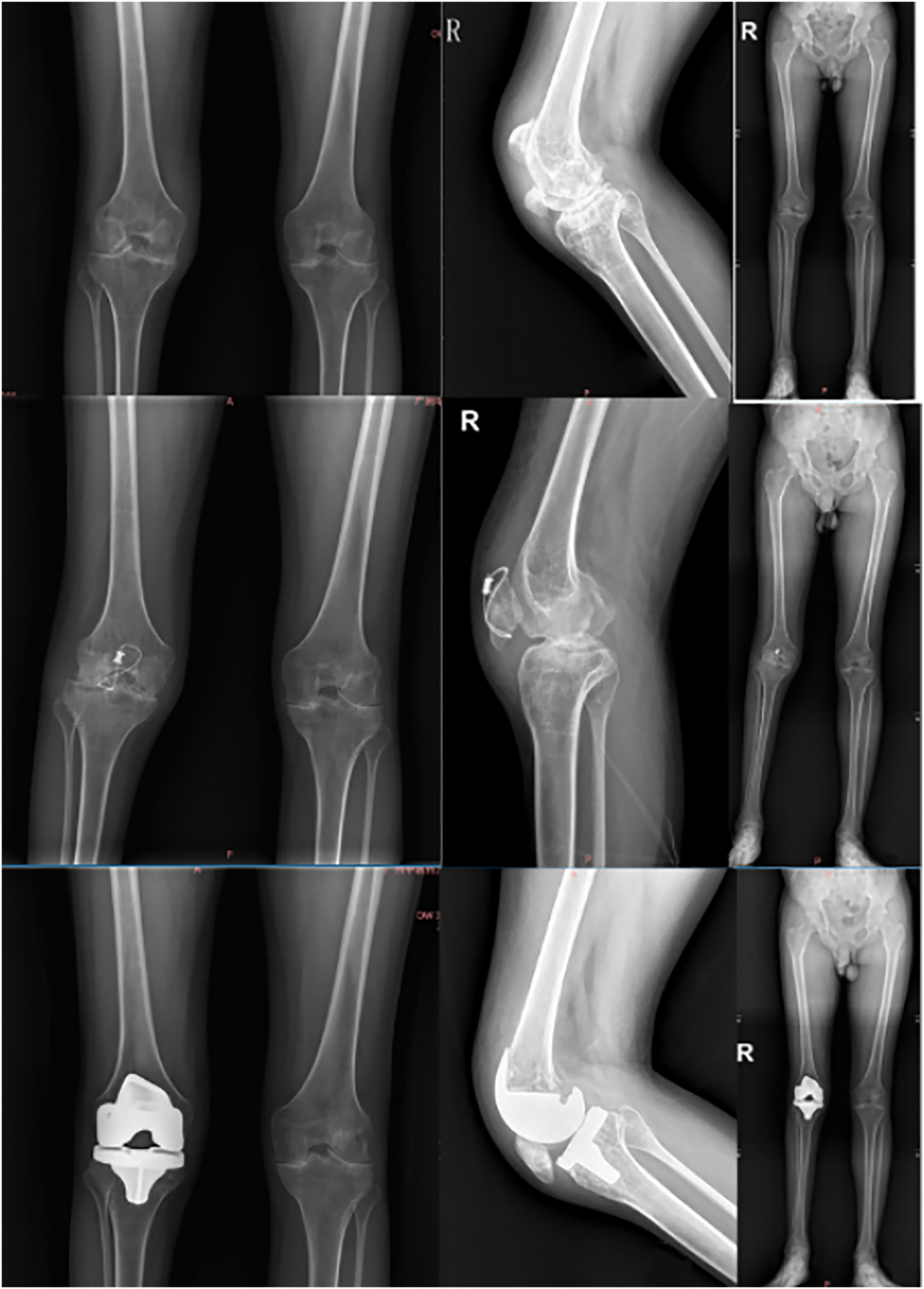
Preoperative and postoperative (2 days) imaging of the patient.

**FIGURE 4:**
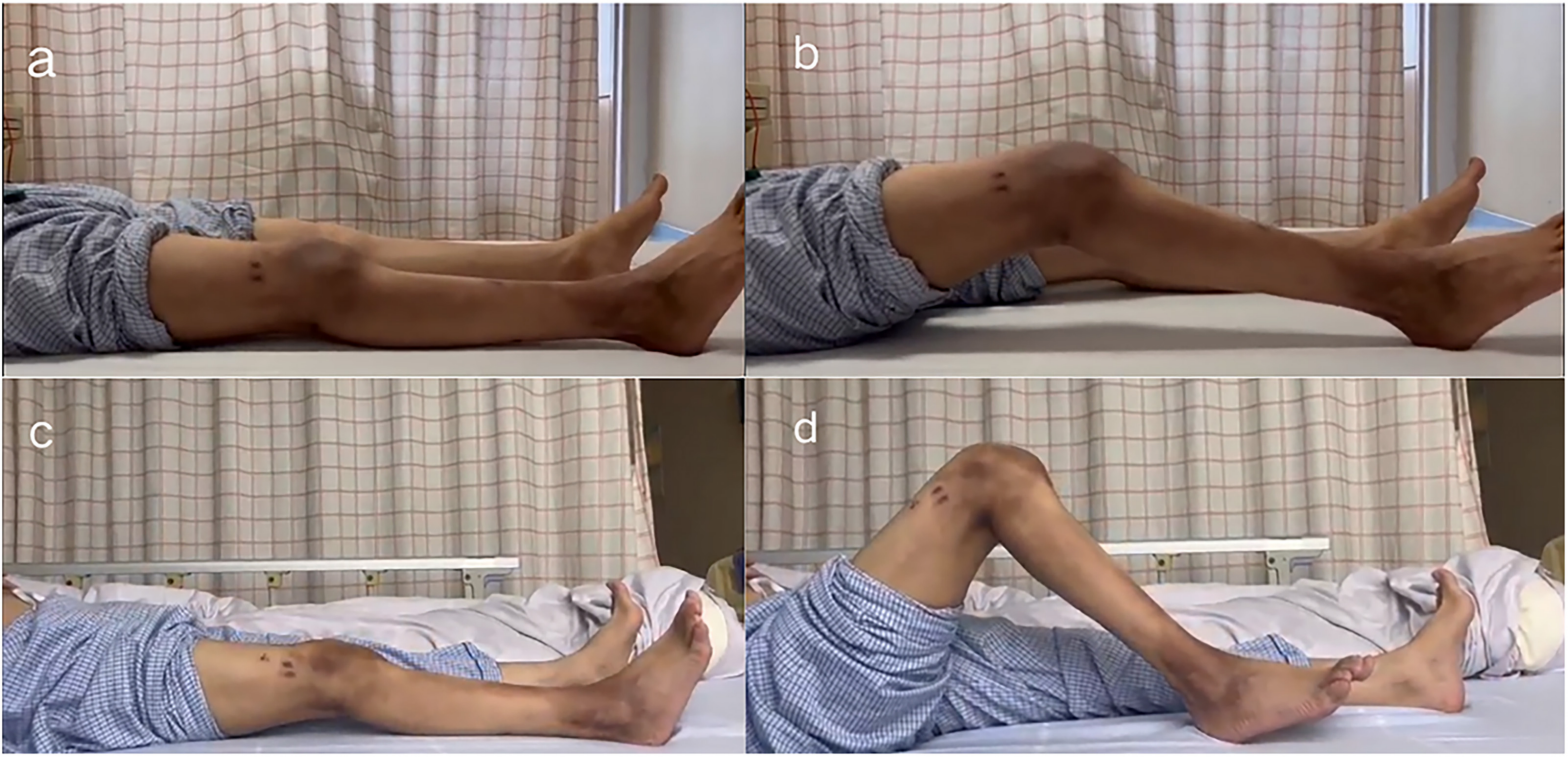
Functional photographs of the patient preoperatively and at 16 months postoperatively. **(a,b)** Preoperative photographs showing a right knee range of motion (ROM) of 20°. **(c,d)** Postoperative photographs at the final follow-up (16 months) demonstrating an improved right knee ROM of 100°.

Preoperative and early postoperative imaging findings are shown in Figure 3. Radiographic evaluation preoperatively and postoperatively confirmed satisfactory prosthetic positioning, partial correction of lower limb alignment, and no evidence of implant loosening. At the final follow-up (16 months postoperatively), the patient achieved full knee extension with a substantial improvement in ROM (0–100°), as illustrated in Figure 4. No complications such as infection, implant loosening, or need for revision surgery were observed.

## Discussion

Patients with hemophilic arthritis and stiff knees frequently encounter inadequate joint exposure during primary TKA, which may result in complications such as flexion–extension gap imbalance, patellar tendon rupture, and ligamentous injury (20). In the present study, a modified quadriceps snip technique was used to obtain sufficient exposure, which allowed satisfactory intraoperative visualization and, combined with targeted postoperative rehabilitation, was associated with favorable functional recovery.

Previous studies have defined a stiff knee as one with an active range of motion (ROM) of less than 50° (21). In hemophilic patients, repeated intra-articular bleeding leads to deposition of hemoglobin and iron within the joint, triggering inflammation and cellular proliferation (22). Iron-laden synovial cells secrete pro-inflammatory cytokines such as interleukin (IL)-1β, IL-6, and tumor necrosis factor–α (TNF-α), which promote synovial hypertrophy, fibrosis, and severe joint deformity (23). These pathological changes accelerate joint degeneration and markedly reduce active ROM. In primary TKA, preoperative ROM has been identified as an independent risk factor for poor postoperative joint mobility (24). The United States Physical Therapy Association (USPTA) indicated that preoperative knee flexion range of motion is one of the most important factors affecting postoperative knee range of motion after arthroplasty (25). In general, the lower the preoperative knee range of motion of patients, the smaller the improvement in postoperative knee range of motion. Y. Liu et al. conducted a retrospective cohort study to analyze the improvement of knee range of motion after total knee arthroplasty (TKA) in 71 male patients (78 knees) with hemophilia and stiff knees. The results showed that the ROM improved from 54.6 ± 32.6° to 80.9 ± 34.5° (13). Mortazavi et al. evaluated the postoperative improvement of knee range of motion in 24 patients (31 knees) with hemophilia and stiff knees undergoing TKA, and reported that the ROM improved from 42.8° to 81.3° (26). The results of the present study demonstrated that the application of the modified quadriceps snip technique in TKA for patients with hemophilia and stiff knees yielded a significant improvement in postoperative knee ROM (from 19.5 ± 11.17° to 80 ± 11.54°), with a greater magnitude of improvement compared with conventional TKA. This finding further reflects the advantages of the modified quadriceps snip technique to a certain extent. Furthermore, Goudie et al. reported that male patients are 2.6 times more likely than female patients to experience limited postoperative ROM (27). Given that the hemophilia population is predominantly male, sex may represent an additional risk factor for postoperative functional impairment.

Currently, a midline knee incision is commonly adopted in hemophilic arthritis patients undergoing TKA. However, conventional approaches may not provide adequate exposure in stiff knees, potentially increasing intraoperative blood loss and complicating postoperative rehabilitation (10). Kuniko Ono et al. demonstrated that a V-Y quadricepsplasty technique can improve exposure while maintaining blood loss comparable to that of conventional approaches (28). Nevertheless, Strauss et al. observed a 9° knee extension lag in patients undergoing V-Y quadricepsplasty despite quadriceps repair, and multiple studies have reported an average postoperative knee extension lag of approximately 7° (29–31). This complication may be related to the absolute lengthening of the quadriceps tendon. Rajgopal utilized lateral retinacular release and various stretching techniques to enhance exposure but still required V-Y quadricepsplasty in four cases because of persistent exposure difficulties, which subsequently resulted in extension deficits (32). Compared with the V-Y quadricepsplasty, the modified quadriceps snip technique improves intraoperative visualization through a specialized incision. The unique suturing method achieves relative lengthening of the distal quadriceps tendon while avoiding absolute tendon elongation. In this case series, although some patients developed postoperative knee extension lag, this functional deficit improved significantly within 6 months postoperatively, with a recovery period shorter than the typical 6–12 months (33). This suggests that the modified quadriceps snip technique may alleviate postoperative knee extension lag by avoiding absolute lengthening of the quadriceps tendon. Meanwhile, in contrast to lateral retinacular release and various stretching techniques, the modified quadriceps snip offers the advantage of providing sustained expansion of the surgical field while improving intraoperative knee flexion.

Tibial tubercle osteotomy (TTO) is another well-established approach to facilitate exposure during TKA. However, this technique complicates the assessment of tibial component rotation and is associated with a complication rate of approximately 9%, including periprosthetic fracture, persistent tibial tubercle pain, and non-union (34). Investigators have further reported that TTO is not suitable for patients with severely limited preoperative range of motion. Accordingly, TTO is not indicated for primary TKA in patients with hemophilic stiff knees. The present case series, to some extent, demonstrates that the modified quadriceps snip technique is appropriate for this patient population with severely restricted preoperative knee motion.

Regarding prolonged drainage and postoperative anticoagulation, patients with hemophilia have a higher bleeding risk than ordinary patients. Moreover, total knee arthroplasty in patients with hemophilic knee stiffness requires extensive intraoperative synovectomy, which creates a large raw wound surface with massive exudation and oozing. Therefore, we adopted the strategy of prolonged closed drainage. Meanwhile, postoperative days 1–7 constitute the critical period for coagulation factor replacement therapy. A 7-day drainage duration is synchronized with the factor protection period, which can effectively cover the high-risk bleeding window. With respect to postoperative thromboprophylaxis, the current guidelines for the diagnosis and surgical management of hemophilia do not recommend routine pharmacological thromboprophylaxis, and mechanical thromboprophylaxis measures are recommended instead (2, 35).

### Limitations

This study has several limitations. First, it was designed as a single-center retrospective study, carrying a potential risk of selection bias. Second, there was no control group consisting of patients with hemophilic arthritis who underwent conventional TKA, and hence no comparative analysis was performed. Third, the sample size was relatively small, and observational non-independence existed owing to the inclusion of 9 patients with 10 affected knees, which limited the external generalizability of the findings.

In addition, blinded outcome assessment was not implemented, and the reporting of postoperative complications was incomplete. Furthermore, this study did not use the Visual Analog Scale (VAS) for independent pain evaluation, nor did it adopt the Medical Research Council (MRC) scale for quantitative grading of muscle strength.

Although the clinical trial design of this study has certain inherent deficiencies and imperfections, the incidence of hemophilic stiff knees is low and clinical cases are extremely scarce. Meanwhile, primary TKA in this patient population is technically demanding and clinically challenging to perform. Therefore, the present study still retains considerable clinical reference value. Further multicenter, large-sample, prospective controlled studies are warranted to verify the long-term efficacy and safety of this modified surgical technique.

## Conclusion

In this small retrospective case series, the modified quadriceps snip technique was correlated with improvements in range of motion (ROM) and clinical outcomes after primary TKA for hemophilic stiff knees. These preliminary descriptive findings suggest that further validation through larger-sample, prospective, controlled studies is still warranted.

## Data Availability

The original contributions presented in the study are included in the article/supplementary material, further inquiries can be directed to the corresponding author.
